# Bone marrow IRF4 level in multiple myeloma: an indicator of peripheral blood Th17 and disease

**DOI:** 10.18632/oncotarget.19907

**Published:** 2017-08-03

**Authors:** Hua Bai, Shuang Wu, Rong Wang, Ji Xu, Lijuan Chen

**Affiliations:** ^1^ Department of Hematology, First Affiliated Hospital of Nanjing Medical University, Jiangsu Province Hospital, Nanjing, China

**Keywords:** multiple myeloma, T-help cells, interferon regulator factor 4, interleukin-17

## Abstract

Interferon regulator factor 4 (IRF4) is characterized to be a member of interferon regulatory family, which is predominantly expressed in bone marrow plasma cells of patients with multiple myeloma (MM). Recent studies indicated IRF4 is critical for T-help cells (Th17) differentiation and interleukin-17 (IL-17) secretion. Here, a total of 58 MM patients were enrolled in this study, the proportions of Th17 cells and T regulatory (Treg) cells in peripheral blood mononuclear cells (PBMCs) were determined by flow cytometric analysis. Immunohistochemistry was employed to detect the IRF4 expression in bone marrow. Herein, we observed a significant increase of IRF4 in bone marrow accompanied with a notable up-regulation of Th17 cells in PBMC within MM patients compared with healthy donors. Furthermore, the proportions of Th17 cells and serum IL-17 levels were higher in patients with stage III than stage I & II MM patients, and those parameters were positively correlated with the expression of IRF4 in these cases. These results for the first time indicate that a crosstalk between IRF4 and Th17 cells is associated with MM prognosis, and IRF4 may be served an important target for MM immunotherapy.

## INTRODUCTION

Multiple myeloma (MM), also known as plasma cell myeloma, is characterized by excess bone marrow plasma cells, monoclonal paraproteins, bone lytic lesions, renal disease and immunodeficiency [1]. Around the world, MM is the second most frequent hematologic malignancy after non-Hodgkin lymphoma [2].

Interferon regulatory factor (IRF) belongs to the nuclear transcription factors, named by its regulation of interferon [3]. IRF families, including more than 10 members, are identified to have recognition of domain amino terminal of DNA with specific sequences and binding function. Functionally, IRF not only regulates cell response to interferon, but also plays a pivotal role in cell proliferation, apoptosis, oncogenic conversion susceptibility and T cell immune responses [3]. Multiple myeloma oncogene 1 (MUM1) / Interferon regulatory factor 4 (IRF4) is one of interferon regulatory factors, which has been shown to be an important transcription factor and is involved in the negative regulation of Th17 cells differentiation and the production of Th17-related cytokines, including IL-17, IL-21 and IL-22 [4, 5]. Mechanically, IRF4 may play a key role in the IL-21-mediated Th17 cells differentiation by influencing the balance of RORα, RORγ and Foxp3 [6]. In plasma cells, IRF4 is also known to be an important modulator for their differentiation [7]. Moreover, IL-1 drives the early differentiation of Th17 cells by regulating the expressions of IRF4 and RORγt [8]. Therefore, mounting evidence support that IRF4 plays a role in the differentiation of Th17 cells.

Th17 cells are recently identified to participate in bacterial infections, and numerous immunologic diseases [9]. The emblematic Th-1 or Th-2 lineages secrete IL-17 rather than IL-4 or IFN-γ [10]. A recent study indicated that IL-17 plays a critical role in the control of the phenotype switch from Th-1 to Th-17, and leads to lytic bone disease in MM [11].

Immunotherapy has emerged as a promising approach for MM treatment recently, but it still lacks targeted specifically and optimized drugs [12]. It is generally accepted that Th17 cells and its relevant cytokines are critical components in the autoimmunity. IRF4 is recognized as an important modulator for the differentiation of Th17 cells. Therefore, defining the relationship between IRF4 and Th17 cells may shed a light for the treatment of MM.

## RESULTS

### IRF4 is highly expressed in MM patients and associated with the MM clinical stages

**Figure 1:**
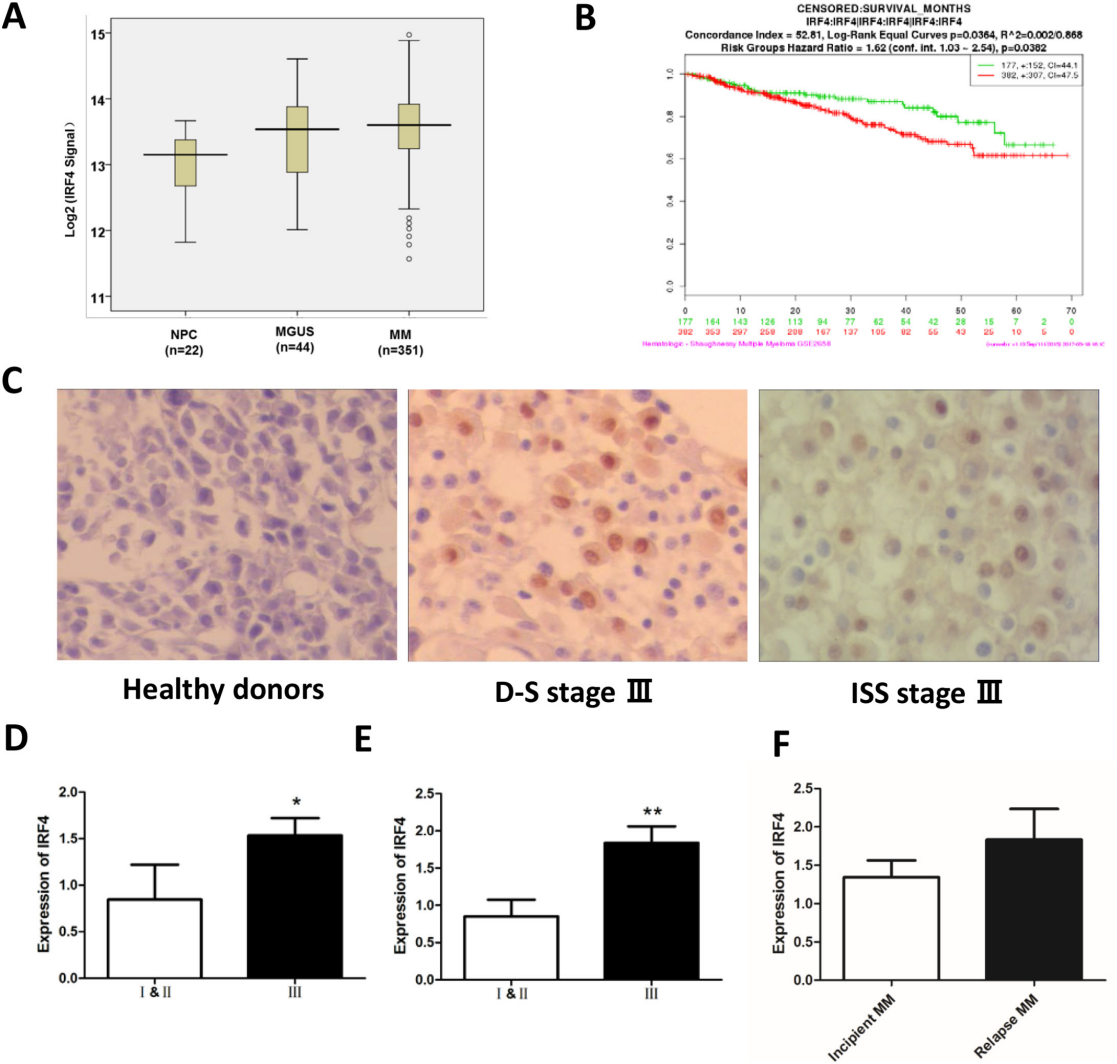
IRF4 is highly expressed in MM patients and associated with the MM clinical stages **(A)** Different *IRF4* expression levels in normal plasma (NPC) cells, MGUS plasma cells and MM plasma cells from the University of Arkansas TT2 cohort (*p* < 0.001). **(B)** Kaplan-Meier analysis and log-rank tests were used to evaluate whether *IRF4* expression level was associated with survival months in MM patients (*p* < 0.05). **(C)** The expression level of IRF4 was observed by immunohistochemical staining analysis in myeloma cells (400×); the expression of IRF4 was negative, the nucleus was not dyed in normal controls; the expression of IRF4 was positive, the nucleus was stained in D-S stage III stages; the expression of IRF4 was positive, the nucleus was stained in ISS stage III stages. **(D)** The expression of IRF4 in D-S stage III were significantly higher than those in patients with stages I & II (*p* = 0.024). **(E)** The expression of IRF4 in ISS stage III were significantly higher than those in patients with ISS stages I & II (*p* = 0.002). **(F)** The expression of IRF4 in incipient MM and relapsed MM patients.

To evaluate the expression of IRF4 in MM patients, we analyzed public gene expression data of purified plasma cells from MM patients (*n* = 351) compared with normal plasma cells (NPC) (*n* = 22) or monoclonal gammopathy of undetermined significance (MGUS) cells (*n* = 44). Statistical comparison of the *IRF4* expression levels in the graph showed that significant up-regulation of *IRF4* is obvious in 351 MM patients compared with NPC and MGUS (*p* < 0.001, Figure 1A). Moreover, we investigated the correlation between *IRF4* expression and survival. Survival months were longer in patients with low *IRF4* expression than in those with high *IRF4* expression (*p* < 0.05, Figure 1B). Next, we measured the IRF4 expression in bone marrow tissues of 58 MM patients and healthy donors by immunohistochemistry. The results showed that 38 cases of MM patients exhibited a distinct increase of IRF4 expression (+∼+ + + +), the positive rate was 65.5% (38/58), including 12 cases (+), 15 cases (+ +), 6 cases (+ + +), 5 cases (+ + + +). IRF4 is found to be expressed in MM cell nucleus, and not observed in lymphocytes, macrophages, and neutrophils (Figure 1C). We then analyzed the correlations between the expression of IRF4 and the clinical parameters. No association was found between the expression of IRF4 and the clinical parameters, such as gender, age, proportion of plasma cells, blood sedimentation (ESR), hemoglobin (Hb), C-reactive protein (CRP), lactate dehydrogenase (LDH), serum creatinine (sCr) and serum albumin (ALB). However, the expression of IRF4 was correlated with aggressive clinical pathological parameters, such as Durie-Salmon (D-S) and International Staging System (ISS) disease stages. In D-S installment, 38 patients were IRF4 positive, including 5 at stages I & II, 33 at stage III; 20 patients were negative IRF4, including 8 at stages I & II, 12 at stage III. In ISS installment, 38 patients were IRF4 positive, including 12 at stages I & II, 26 at stage III; while 20 patients were IRF4 negative, including 15 at stages I & II, and 5 at stage III. Statistical analyses showed that the expression of IRF4 in the MM stage III patients were significantly higher in patients with disease stages I & II (D-S stage: *p* = 0.024, Figure 1D; ISS stage: *p* = 0.002, Figure 1E). However, the IRF4 expression is not significantly different between incipient MM and relapsed MM patients (Figure 1F).

### Proportion of Th17 cells is associated with the MM clinical stages

**Figure 2:**
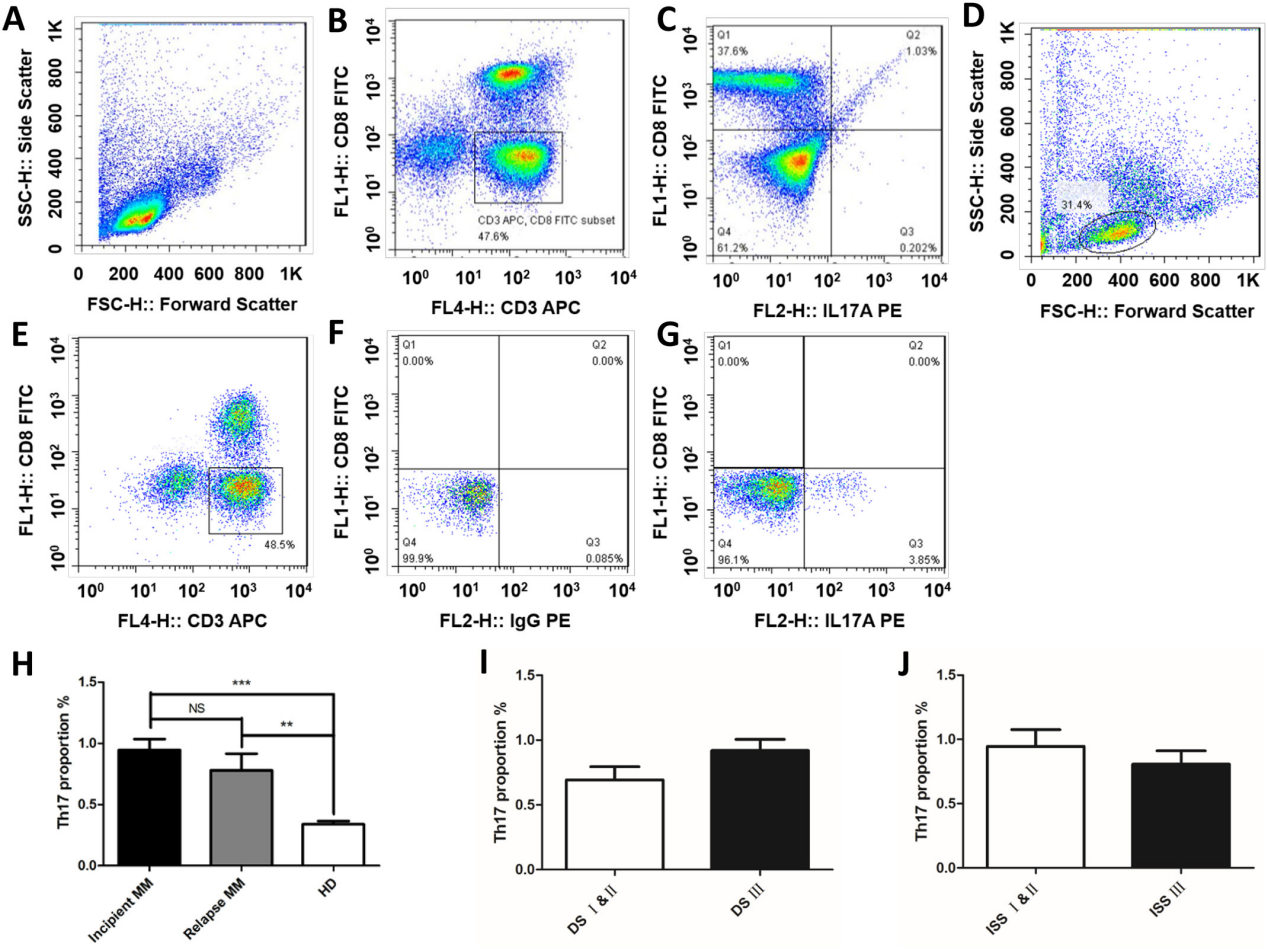
Th17 cells is correlated with the MM clinical stages The ratios of Th17 cells in peripheral blood (CD3^+^ CD8^-^ IL-17A^+^) were detected by flow cytometric analysis in MM patients and healthy donors. The proportions of Th17 cells of normal people **(A)** the gate for lymphocytes was set by the FSC and SSC. **(B)** CD3^+^CD8^-^ lymphocytes were selected by CD3 and CD8 door. **(C)** The ratio of CD3^+^ CD8^-^ IL-17A^+^ in CD3^+^ CD8^-^ lymphocytes. The proportions of Th17 cells of MM patient **(D)** the gate for lymphocytes was set by the FSC and SSC. **(E)** CD3^+^CD8^-^ lymphocytes were selected by CD3 and CD8 door. **(F)** The same type of control chart for IL-17A. **(G)** The ratio of CD3^+^ CD8^-^ IL-17A^+^ in CD3^+^ CD8^-^ lymphocytes. **(H)** The proportions of Th17 cells in CD3^+^CD8^-^ lymphocytes were detected by flow cytometric analysis in MM patients, the results showed that Th17 cells in the incipient and relapsed patients were significantly higher than normal (**p < 0.01, ***p < 0.001), Th17 cells in the incipient patients were higher than that in patients with recurrence, but there was no significant difference between them (*p* > 0.05). The percentages of Th17 cells in DS stage and ISS stage patients **(I)** DS stage; **(J)** ISS stage.

Flow cytometric analysis was performed to detect the Th17 cells in peripheral blood (CD3^+^ CD8^–^ IL-17A^+^) accounted for the proportion of CD3^+^ CD8^-^ lymphocytes in 58 MM patients and 20 healthy donors (Figure 2A-2G), the Th17 cells in the incipient and relapsed MM patients were significantly higher than healthy donors (HD) (0.95 ± 0.10% *vs.* 0.34 ± 0.03%, *p* < 0.001; 0.78 ± 0.14% *vs.* 0.34 ± 0.03%, *p* < 0.01; Figure 2H). The levels of Th17 cells in the incipient patients were higher than those relapsed samples, but it lacked statistical significance (0.95 ± 0.10% *vs* 0.78 ± 0.14%, *p* > 0.05). We correlated the proportion of Th17 cells with clinical indicators in the 38 incipient MM samples, there was no significance in the sex, age, Ig subtype, stage, proportion of plasma cells, ESR, Hb, CRP, LDH, sCr and ALB. In addition, the percentage of Th17 cells did not show a difference between stage III and stages I & II in both DS and ISS classifications (Figure 2I & 2J).

### Elevated levels of serum IL-17 in MM patients

**Figure 3:**
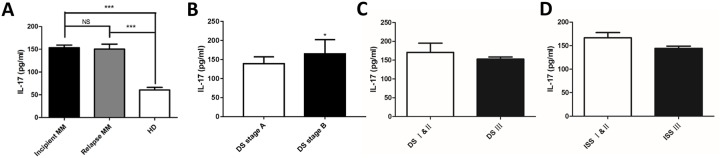
Elevated levels of serum IL - 17 in MM patients The levels of IL-17 in serum were detected by ELISA in MM patients and healthy donors. **(A)** The levels of IL–17 in the incipient and relapsed patients were higher than healthy donors (***p < 0.001). **(B)** The levels of IL-17 in DS stage B were higher than those in stage A (*p < 0.05). The IL-17 levels in DS stage and ISS stage patients **(C)** DS stage; **(D)** ISS stage.

ELISA was used to assess the serum levels of IL-17 from peripheral blood of MM patients and healthy donors, the secreted IL-17 was significantly higher in the incipient and relapsed MM patients than those from healthy donors (153.66 ± 34.01 vs. 60.67 ± 24.90 pg/ml, *p* < 0.001; 150.47 ± 46.90 vs. 60.67 ± 24.90 pg/ml, *p* < 0.001; Figure 3A), but there was no significant difference between the incipient and relapsed MM patients (153.66 ± 34.01 *vs.* 150.47 ± 46.90 pg/ml, *p* > 0.05), which is consistent with the levels of Th17 shown in the Figure 2H. We analyzed the correlations between the levels of IL-17 and clinical indicators from these 38 incipient MM patients, the levels of IL-17 in group A (serum creatinine ≤ 176.8 μmol/L; *n* = 22) are lower than group B (serum creatinine ≥ 176.8 μmol/L; *n* = 16) of MM patients according the DS stages (146.21 ± 30.90 pg/ml *vs.* 173.19 ± 37.19 pg/ml; *p* < 0.05, Figure 3B). Consistently, there was no significance between the levels of IL-17 with sex, age, stage, ESR, Hb, CRP, LDH, and ALB (Table 1). Moreover, no significant difference was found in IL-17 levels between DS stage III and DS stages I & II of MM patients. Similarly, IL-17 levels were not significantly different between ISS stage III with ISS stages I & II of MM patients (Figure 3C & 3D).

**Table 1:** Correlations of clinical parameters with IL-17 in 38 incipient MM patients

Clinical index			*p* value
Gender	Male (26)	Female (12)	0.333
Age (years)	<60 (22)	≥ 60 (16)	0.265
DS stages	I & II (9)	III (29)	0.244
ISS stages	I & II (17)	III (21)	0.084
**Laboratory examination**			
sCr (μmol/L)	<176.8 (22)	≥ 176.8 (16)	0.036
CRP (mg/L)	<10 (31)	≥ 10 (7)	0.058
ESR (mm/H)	<100 (14)	≥ 100 (24)	0.932
ALB (g/L)	<35 (31)	≥ 35 (7)	0.837
LDH (U/L)	<270 (32)	≥ 270 (6)	0.932

*p* < 0.05 was considered to reflect statistical significance. sCr, serum creatinine; CRP, C-reactive protein; ESR, erythrocyte sedimentation; ALB, serum albumin; LDH, lactate dehydrogenase.

### Proportion of Treg cells is associated with elevated levels of serum calcium

**Figure 4:**
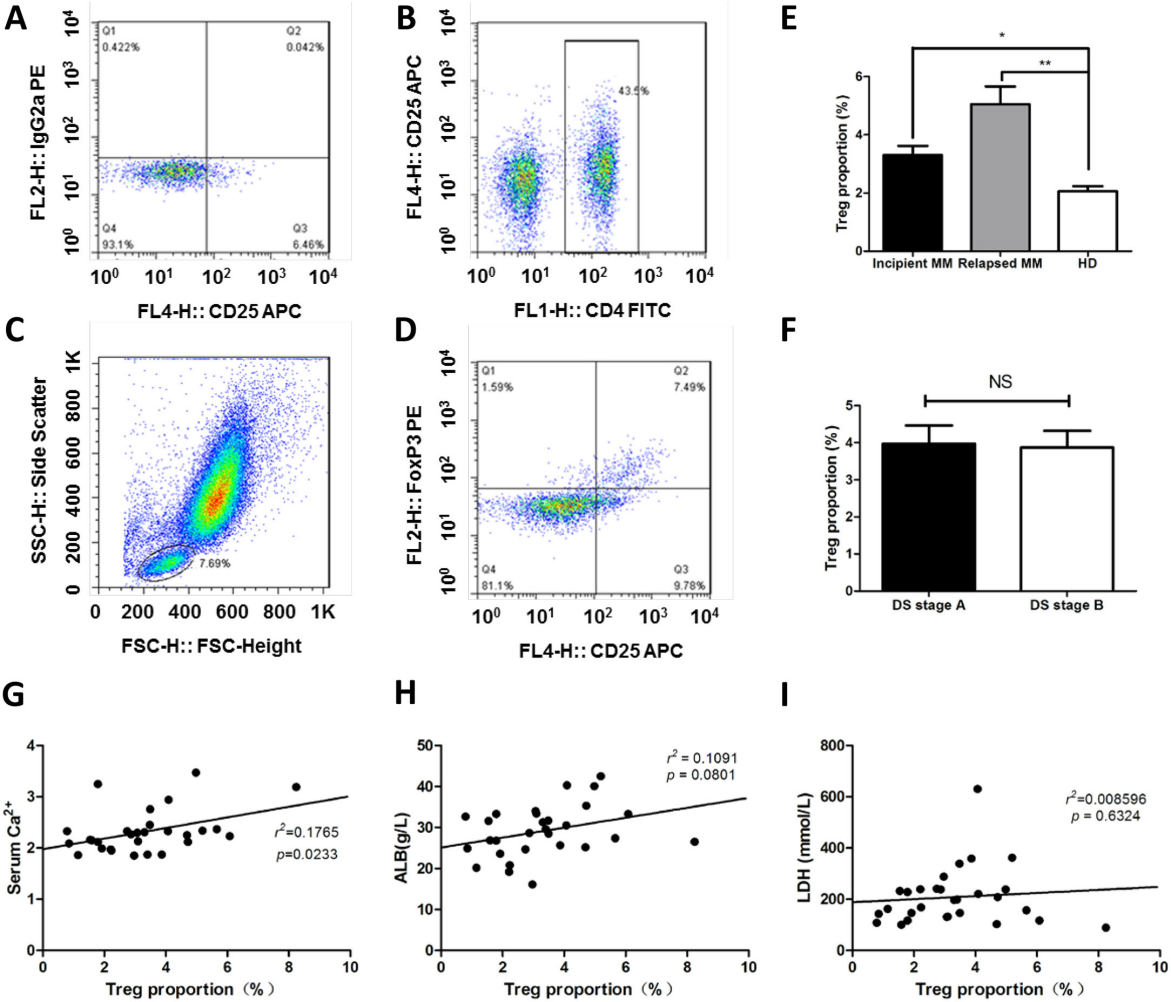
The proportions of Treg cells is increased in MM patients The ratios of Treg cells in peripheral blood (CD4^+^CD25^+^FoxP3^+^) were detected by flow cytometry in MM patients. **(A)** The gate for lymphocytes were set by the FSC and SSC. **(B)** CD4+ lymphocytes were selected by CD4 and CD25 door. **(C** & **D)** the ratio of CD4^+^CD25^+^FoxP3^+^ Treg cells in CD4^+^ lymphocytes. **(E)** The proportions of Treg cells in CD4^+^ lymphocytes were detected by flow cytometry, the results showed that Treg cells in the incipient and relapse patients were significantly higher than normal (3.23 ± 1.69% *vs.* 2.16 ± 0.71%, 5.06 ± 2.41% *vs.* 2.16 ± 0.71%, *p* < 0.01), Treg cells in the relapsed patients were higher than that in incipient patients (5.06 ± 2.41% *vs.* 3.23 ± 1.69%, *p* < 0.01). **(F)** The percentage of Th17 cells in DS stage. **(G-I)** Correlations of clinical parameters with Treg cells in incipient MM.

The balance between Treg and Th17 cells acts as an essential prerequisite for regulating anti-tumor immunity in MM [13]. So the proportions of Treg cells (CD4^+^CD25^high^FoxP3^+^) in peripheral blood were also detected in healthy donors (*n* = 20) and MM patients (*n* = 58) by flow cytometric analysis (Figure 4A-4D), the Treg cells in the incipient and relapsed MM patients were significantly higher than healthy donors (3.23 ± 1.69% *vs.* 2.16 ± 0.71%, *p* < 0.05; 5.06 ± 2.41% *vs.* 2.16 ± 0.71%, *p* < 0.01; Figure 4E), but there was no difference between DS stage A and DS stage B groups (Figure 4F). We also correlated the proportion of Treg cells with clinical indicators in the 38 incipient MM samples (Figure 4G-4I), and the proportion of Treg cells was positively related to the expression levels of serum calcium (*r*^*2*^ = 0.1765, *p* = 0.0233, Figure 4G).

### The levels of Th17 and IL-17 are positively related to the expression of MUM1/IRF4

**Figure 5:**
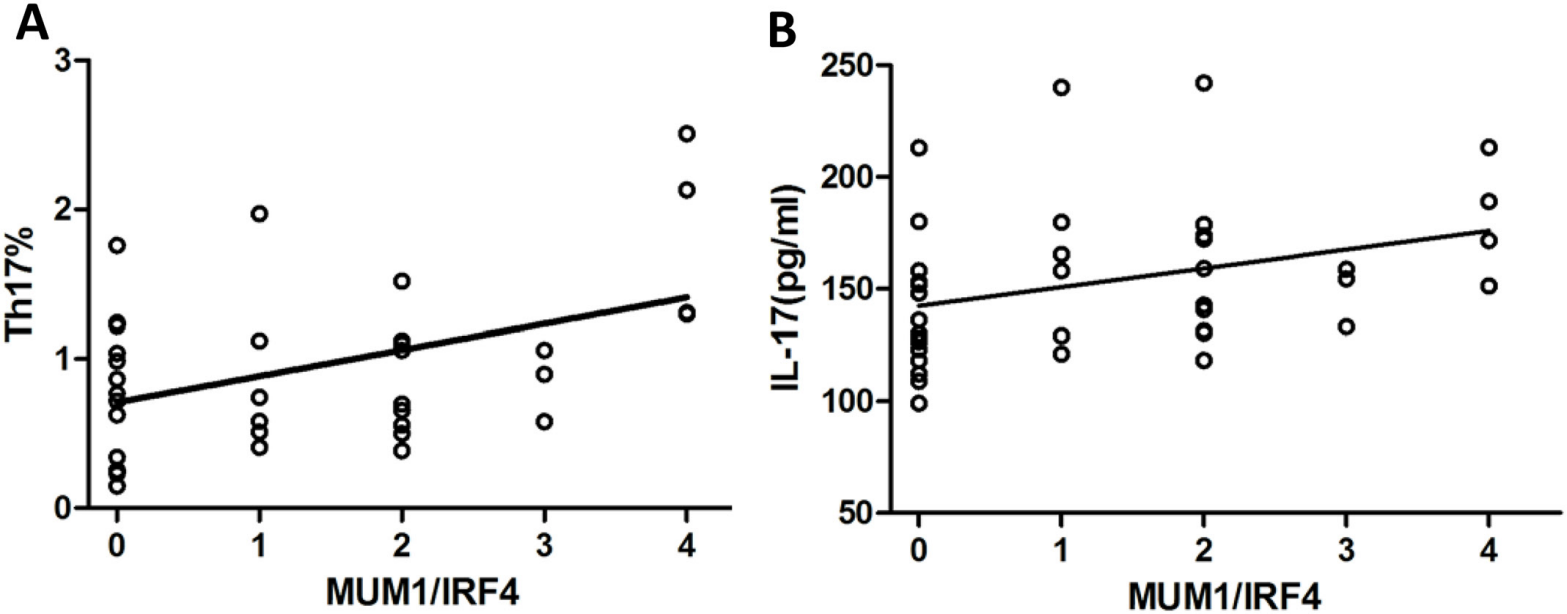
The levels of Th17 and IL-17 are positively related to the expression of MUM1/IRF4 The correlations between Th17, IL-17 and IRF4 were analyzed by Pearson′s correlation. **(A)** The proportions of Th17 cells were positively related to the expression of MUM1 / IRF4 (*r* = 0.379, *p* = 0.027). **(B)** The levels of IL-17 in serum were positively related to the expression of MUM1 / IRF4 (*r* = 0.406, *p* = 0.011).

To determine the correlation between Th17 and IRF4 in MM patients, we analyzed the proportions of Th17 cells and levels of IL-17 in 38 incipient MM patients. As shown in Figure 5, both proportions of Th17 and IL-17 were positively related to the expression of MUM1/IRF4 (*r* = 0.379, *p* = 0.027, Figure 5A; *r* = 0.406, *p* = 0.011, Figure 5B).

## DISCUSSION

Abnormal cytogenetic is one of the important factors influencing the prognosis of MM, it involves in a series of changes in the bone marrow microenvironment for MM tumorigenesis and immune regulation. Many studies have reported that MM patients have an immunodeficiency, which is worth of further study to its clinical relevance [13]. The present study is focused on the correlation between Th17 cells and IRF4 in MM patients. The Th17 cell is a new type of CD4^+^ T helper cells, known to protect the body against fungi and parasitic infections and involved in autoimmune and the body's inflammatory response [13]. A previous study has found that Th17 cells proportion was associated with ISS stages, levels of LDH, and serum creatinine, indicating that Th17 cells proportion can be reduced after treatment [14]. However, our current study did not support this conclusion. Th17 cells proportion was not associated with ISS stages, levels of LDH, ALB, ESR and serum creatinine.

IL-17, one of the most important cytokines secreted by Th17 cells, is highly expressed in many cancers, and some autoimmune or infectious diseases [15, 16]. It was reported that the levels of IL-17 in incipient and relapsed MM patients were obviously higher than those in healthy donors, but there is no significant difference between incipient and relapsed MM patients. A significant increase in Th17/IL-17 expression in MM patients were presented, which could be due to the fact that the interactions between myeloma cells and bone marrow microenvironment. The increases of Th17/IL-17 are crucial in promoting tumorigenesis, drug resistance, angiogenesis, and bone destruction through secretion of adhesion molecules and cytokines with these malignancy cells. Previous studies also showed that the IL-17 level was high in MM patients, which is closely related to bone lesions and tumor cell growth [17–19]. It has also been found that the serum TNF-α and VEGF in MM patients was significantly higher than healthy donors. TNF-α and VEGF are related to the levels of IL-17, which indicate that IL-17 may be involved in the progress of angiogenesis, and the progress of the disease [20]. Kim *et al.* found that gene polymorphisms in IL-17 and IL-17R were associated with the end-stage renal disease (ESRD), their study indicated that both IL17E rs10137082 and IL17RA rs4819554 are highly expressed in patients with ESRD, which revealed the relationship between IL-17 and kidney damage [21]. We also analyzed the correlation between the levels of IL-17 and clinical stages in 38 cases incipient MM patients, then we found that levels of IL-17 in the DS staging group A patients was lower than the patients in group B, instructing the correlation between renal damage and elevated levels of IL-17 in patients. Consistent with previous reports, we revealed that the relationship between Th17/IL-17 and disease stage, and speculated that the mechanisms of immune disorders and inflammatory reaction in the course of the MM, nevertheless further study need to expand the sample size and ascertain the critical role of Th17/IL-17 as a prognostic maker for MM.

Treg cells are responsible for the control of autoimmune phenomena, such as maintaining immunologic self-tolerance, anti-myeloma immunity, and undermining anti-infectious [22, 23]. Previous studies have shown that the proportion of Treg cells is increased MGUS and MM patients compared with healthy donors, which is consistent with our results [24, 25]. Bryant *et al.* found obviously higher proportion of Th17 cells and lower proportion of Treg cells in the long-term survivors (LTS-MM) group compared with other MM group [26]. However, we found there is no difference of Treg/Th17 between MM patients and healthy donors. We also analyzed the correlations between the proportion of Treg cells and clinical indicators in 38 cases incipient MM patients, and then we found the proportion of Treg cells was positively related to the expression levels of serum calcium, which is consistent with previous study [27].

MUM1/IRF4 influences the differentiation of T cell in different periods by controlling the expression of cytokines and apoptosis. Brustle *et al.* showed that MUM1/IRF4 plays an important role in the differentiation of T helper cells; it can not only affect the growth of Th2 cells, but also influenced the differentiation of Th17 cells [4]. Biswas *et al.* investigated that the activation of serine-threonine kinase Rho-associated, coiled-coil–containing protein kinase 2 (ROCK2) made IRF4 phosphorylation and regulated the combining ability of IRF4 with IL-17 and IL-21 in the differentiation of Th17 [28]. Here, we found that the positive rate is 65.5% in MM patients with bone marrow biopsy by immunehistochemical analysis. Knocking down of IRF4 could cause the rapid death of MM cells, prompting IRF4 is a characteristic expression of myeloma cells [29], and the dysregulation of IRF4 could contribute to malignant transformation, indicating IRF4 is also a precursor state for the development of MM [30]. Most of literatures suggested IRF4 up-regulation is correlated with poor prognosis [31, 32], we also analyzed public gene expression data and the correlations between the expression of IRF4 and the clinical parameters of MM patients, and found IRF4 in patients with end-stage disease was significantly higher than those patients with early-stage disease, which indicated that the increased expression of IRF4 is associated with poor prognosis. Some studies showed that IRF4 is an important transcriptional factor in the differentiation of Th17 cells and the decrease of IRF4 prevents the differentiation of Th17 cells [4, 5]. Similarly, we found the correlations between IRF4 expression and proportion of Th17 cells or the levels of IL-17 are positive, prompting IRF4 participates in the differentiation of Th17 cells and the secretion of IL-17. The immune system plays an important role in growth of myeloma cells, but the mechanisms controlling T helper cell lineage by IRF4 in MM is unknown. The immune therapy was followed with interests and the research for MM immune mechanism is especially valuable.

Our findings illustrated that the levels of MUM1/IRF4, Th17 and IL-17 are increased in MM patients, and related with the stage of disease. The peripheral blood Th17 cells numbers and the serum levels of IL-17 were positively related to the intensity of MUM1/IRF4 expression. These results raised the possibility that MUM1/IRF4 acts as an effector in MM by promoting Th17 amplification and IL-17 secretion.

## MATERIALS AND METHODS

### Patients and clinical features

58 MM patients were collected from January 2012 to March 2014 in the First Affiliated Hospital of Nanjing Medical University, diagnosis were conformed to the 2008 World Health Organization (WHO) criteria, and curative effect standard was approved by the International Working Group the curative effect of standard. The subjects were divided into incipient (38 cases) and relapsed (20 cases) groups. A total of 38 of the subjects were male, 20 of them were female, with median onset age of 58.2 years (40 ∼ 80 years). The patients were also classified depending on the types of M protein secretion. IgG isotype was present in 41.3% (24/58), whereas 18.9% (11/58) of them had IgA, 1.7% (1/58) had IgD, κ light chain type 12.0% (7/58), λ light chain type 20.6% (12/58) and non-secretion 5.1% (3/58). According to the Durie-Salmon (D-S) staging system, 4 patients had stage I, 9 had stage II, and 45 had stage III. According to International staging system (ISS), 7 had stage I, 23 had stage II, and 28 had stage III. The healthy controls were classified into two groups, including 12 cases of male, 8 cases of female; the average age was 54, with no history of autoimmune disease, tumor, or recent infection. The selection of treatment regime for primary induction therapy was based on MM patients’ characteristic, such as the risk of toxicity, the patients’ intension, and the capacity of patients to tolerate drugs. 43.1% (25/58) patients were treated with bortezomib-based regimen, 56.8% (33/58) patients were treated with thalidomide-based regimen.

### Gene expression profiling (GEP) and data analysis

Gene Expression Omnibus (GEO) data were carried out to examine the expression of *IRF4* in normal plasma (NP), monoclonal gammopathy of undetermined significance (MGUS) (GSE5900), and MM (GSE2658) plasma cells. The plasma cells were determined using the Affymetrix U133Plus2.0 microarray (Affymetrix, USA), which were performed as previously described [33].

### Flow cytometric analysis

Ficoll was utilized to diluteperipheral blood in a 15 mL conical tube and centrifuged at room temperature, then we collected the mononuclear cells from the plasma/Ficoll interface into a new sterile conical tube, raised the cells with RPMI-1640 memdium (20%FBS+ Leukocyte Activation Cocktail). Next, the percentages of Th17 cells were identified and counted by anti-CD3, and anti-CD8 antibodies. PBMC from MM patients were first washed by PBS for twice and then put with anti-CD3 APC and anti-CD8 FITC mAb (BD Biosciences, USA) for 15 minutes at room temperature. After washings with PBS/BSA, the percentages of positive cells were calculated by FACSCalibur (BD Biosciences, USA).

### Measurement of cytokines by ELISA

The serum was separated and stored at -80°C until assayed. The levels of IL-17 were detected using an ELISA (R&D system, USA) kit according to the manufacturer’s recommendations. IL-17 was detected using IL-17 antibody and quantified at 450 nm with a reference wavelength of 655 nm. Each sample was analyzed in duplicate, performed three independent experiments.

### Immunohistochemistry

The BM tissues were harvested and fixed in 10% formaldehyde, and antigen retrieval was performed in EDTA-containing antigen retrieval buffer (pH = 8.0) in 95°C followed by 3 % H_2_O_2_ incubation for 30 min. After blocking by goat serum for 10 min, Anti-IRF4 (Novocastra, UK) monoclonal antibody was incubated over night at 4°C, the secondary antibodies were incubated for 30 min at room temperature before visualization by DAB reagent. The intensity standards were: pain 0 point; shallow yellow : 1 point; pale yellow : 2 point; sepia: 3 point. Calculate the products of stain intensity and positive cell quantities, results are determined with principle as follows : 0∼3 points is ( - ), 4∼5 points is ( + ), 6∼9 points is ( ++ ), 10∼12 points is ( +++ ).

### Statistical analysis

The statistical analysis was performed with the GraphPad Prism 5 (GraphPad, Inc., USA). Correlations of IRF4 with other clinical parameters were performed using Spearman’s correlation. Student’s t-test was used for between-group comparisons of discrete clinical parameters. Survival curves were plotted using Kaplan-Meier method and log-rank test was applied for comparison. Data of different stages (D-S and ISS) were compared using chi-square analyses. *p < 0.05 were considered to reflect statistical significance.
